# Tourism and Green Development: Analysis of Linear and Non-Linear Effects

**DOI:** 10.3390/ijerph192315907

**Published:** 2022-11-29

**Authors:** Yuanyuan Wu, Zhanhua Jia, Tingting Yu

**Affiliations:** 1 School of Tourism Management, Hubei University, Wuhan 430062, China; 2 School of Economics, Faculty of Economics, Liaoning University, Shenyang 110136, China; 3 College of Education for the Future, Beijing Normal University, Zhuhai 519000, China

**Keywords:** tourism, green development efficiency, super-efficiency SBM, non-linear, dynamic panel threshold model

## Abstract

Clarifying the relationship between tourism and green development is conducive to promoting the harmonious coexistence of tourism industry benefits and economic and environmental systems. The externalities of tourism on economies and the environment have sparked numerous fascinating academic research debates; however, few studies have considered the impact of tourism on green development that balances economic growth and environmental protection. This study selects the green development efficiency measured by the super-efficient SBM model with undesired output as a proxy indicator of green development and adopts the panel data regression model and dynamic panel threshold regression model to investigate the linear impact and non-linear characteristics of tourism on the green development efficiency for 284 cities in mainland China at the prefecture level and above. The main findings are as follows: (1) Although China’s green development efficiency showed an upward trend during the study period, the overall level was not high. (2) Tourism has significantly promoted the improvement of China’s green development efficiency, indicating that tourism has become an effective driver of China’s economic green transformation. (3) This type of positive promotion of green development by tourism has a non-linear threshold characteristic, which means that, with the continuous improvement of the development level of the tourism industry, after crossing a specific threshold value and entering a higher level of development, the tourism industry will have an increasing marginal impact on the green development efficiency.

## 1. Introduction

In recent decades, climate change and environmental pollution caused by increased energy consumption have been major constraints on the global economy achieving sustainable development. China, which has the most complex natural environment and social and economic conditions in the world, has also faced the dual challenges of tightening resource constraints and serious environmental pollution, while its economy has grown rapidly since its reform and opening up. How to manage the relationship between economic development and environmental protection, and achieve a positive interaction, is a real issue for many countries today. Green development, which aims at efficiency, harmony, and sustainability, can break the existing resource and environmental constraints on economic development and achieve a harmonious symbiosis between resource conservation, environmental friendliness, and economic growth [1,2]. Therefore, green development is gradually becoming a global consensus for economic transformation and reconstruction [3], as well as the basis for building an ecological civilisation and the ideal of a beautiful China [1,4].

Green development needs the support of a modern industrial system that adapts to it. As a modern service sector and one of the world’s largest industries, tourism plays a pivotal role in regional economic development because of its ability to absorb foreign exchange, generate income, and increase employment opportunities [5,6,7]. According to the data released by the World Travel & Tourism Council, in 2019, the total global tourism revenue reached 9.2 trillion US dollars and created 334 million jobs; its combined contribution to GDP and employment reached 10.4% and 10.6%, respectively [8]. However, tourism cannot support the economic development of all regions in the long term, especially those destinations with poor economic foundations and economic development heavily dependent on tourism. This can lead to the problem of destination deindustrialisation and the Dutch disease effect, which can harm local economic development [9,10,11]. Furthermore, some studies have proven that the impact of tourism on economic growth is not stably positive or negative, but with the improvement of the tourism development level, it shows non-linear characteristics [12,13,14].

In addition to the economic impact, the environmental impact of tourism has also received extensive academic attention. Generally, tourism is accused of negatively impacting the environment in the form of greenhouse gas emissions, mainly from the direct or indirect use of fossil fuels by tourism activities [15,16,17]. Air pollution, water pollution, solid waste and garbage, and soil erosion are also manifestations of adverse environmental impacts in the development of tourism [18,19]. However, tourism can also be a tool to combat environmental degradation, especially well-managed tourism, which can have a positive impact on the environment through the use of environmentally friendly technologies and modes of transport [20]. In addition, tourism may also have a non-linear effect on environmental degradation. Considering the dependence of tourism on the environment, with the development of tourism at a certain stage, the tourism industry and governments will also take measures to alleviate its negative impact on the environment [21,22].

In the context of the reality of the green transition and the research base on the economic and environmental impacts of tourism, the following questions arise: What role does tourism play in green development that balances economic growth and environmental protection? Is the marginal effect of tourism on green development as non-linear as its impact on economic and environmental factors? Research on these issues will contribute to enriching research on tourism externalities and the research framework of its effects on green development, establish a tourism industry development model from the perspective of green development, and promote the coordinated coexistence of tourism industry benefits and economic and environmental systems.

While the existing literature has extensively explored the impact of tourism on the economy or the environment, few studies have integrated these two directions into a unified research framework to answer the question of how tourism affects green development. In particular, there is a lack of studies exploring the relationship between tourism and green development based on a non-linear perspective. Given the uncertainty in the mechanism of tourism’s effect on the economy or the environment and the heterogeneity of tourism development stages among tourism destinations, the impact of tourism on green development may be non-linear. In addition, previous studies mostly tested the non-linear impact of tourism on the economy or the environment by grouping regression, introducing square terms, or using a traditional threshold regression model [14,19,22,23,24]; however, the first two methods often have the problem of subjectivity and high collinearity. Although the traditional threshold regression model can overcome the defects of the first two methods, it is also limited by maintaining the assumption of the exogeneity of the explanatory or threshold variables [25]. As some studies provide evidence of a bidirectional causal relationship between tourism and the economy or the environment [6,19,26], this implies that the tourism variable is endogenous. If the static threshold model is still used to test for non-linear relationships, the estimation results will be biased.

To answer the above research questions and bridge the gaps in existing research, this paper analyses the mechanism of tourism’s impact on green development and proposes corresponding hypotheses. On this basis, the green development efficiency measured by the super-efficient SBM model with undesired outputs is used as a proxy for green development, and a panel data regression model and dynamic panel threshold analysis are employed to empirically test how tourism affects green development. The analysis is conducted using China as a case study, as a civilised country with green development policies and as one of the most popular tourist destinations in the world. This paper contributes to the existing literature as follows: First, this study integrates the economic and environmental impacts of tourism into a unified research framework, explores the mechanisms by which tourism affects green development, and provides empirical evidence on whether tourism promotes green development in tourist destinations. Second, the relationship between tourism and green development is examined from a non-linear perspective, based on the uncertain impact mechanism of tourism on the economy or the environment, as well as the heterogeneity of tourism development stages in specific destinations. Third, a dynamic panel threshold model is employed to explore the non-linear impact of tourism on green development to address potential endogeneity issues and obtain robust estimation results.

The remainder of this article is organised as follows. Section 2 briefly reviews the key literature, analyses the mechanism, and proposes the research hypothesis. Section 3 introduces the empirical methodology, models, and data. Section 4 presents the relevant analysis of the empirical results. Section 5 discusses the empirical results and illustrates the limitations of this paper and future research directions. Section 6 draws the research conclusions.

## 2. Literature Review and Research Hypothesis

### 2.1. Literature Review

#### 2.1.1. Green Development

Green development is a new concept that brings the external environmental cost into the regional economic development system, derived from the concept of sustainable development [2]. It started with the first mention of sustainable development in the report “Our Common Future” by the World Commission on Environment and Development in 1987. Since then, the concepts of green economy, green growth, and green development have been successively proposed to solve the practical needs of development and sustainable problems [4,27,28]. Although green development is similar to sustainable development, the green economy, and green growth in ideology, it has formed a more systematic research system in terms of concept and connotation, and evaluation and measurement methods.

The existing literature explains the concept and connotation of green development from both narrow and broad perspectives. In a narrow sense, green development is interpreted from the perspective of economic growth. It is a comprehensive development model that integrates environmental constraints into the economic growth framework, promotes economic growth and environmental quality through energy conservation and emission reduction, and emphasises the unity and coordination of economic growth and environmental protection [1,2,29]. In a broad sense, green development can be understood from the perspective of sustainable development. It not only aims to enhance economic vitality and improve environmental quality, but also to improve residents’ well-being and social equity, emphasising the symbiosis and coordination between the economic system, social system, and natural system [4,30,31].

For the evaluation and measurement methods of green development, some relevant studies are based on the evaluation framework of sustainable development through building a complex multi-level evaluation index system and using a comprehensive evaluation method, entropy weight method, and DPSIR model to evaluate the level of green development [31,32,33]. However, in the process of building the indicator system, the assessment results of green development may be affected due to its subjectivity. Therefore, there are also studies based on efficiency measurement, taking the green development efficiency as an important indicator to evaluate the green development level, and using data envelopment analysis to calculate the green development efficiency [1,2,3,4,29,30].

#### 2.1.2. The Economic Impact of Tourism

The impact of tourism development on the economy has long been a research topic that has attracted much attention. Regardless of the theoretical analysis of the internal logic or the empirical research, the promotion of economic growth by tourism development has always been the mainstream view of the economic impact of tourism. Theoretically, the related studies are based on the Keynesian Multiplier Effect. Tourism is regarded as part of the exogenous aggregate demand that has a positive impact on regional income and employment through the multiplier effect that is mainly measured by input–output analysis [34,35] and the general equilibrium model [36]. Empirically, most scholars have carried out many explorations of the contribution of tourism to economic growth, especially marked by the tourism-led growth hypothesis (TLGH) proposed by Balaguer and Cantavella-Jordá [37]. Many empirical studies have focused on this topic and have employed time series or panel data econometric models to empirically test the authenticity of the TLGH in a single destination [26,38,39,40] or multiple destinations [6,41,42,43].

However, some studies deny the general validity of the idea that tourism promotes economic growth. The most classic criticisms are the deindustrialisation problem proposed by Copeland and the Dutch disease effect proposed by Chao et al. They generally agree that, although the expansion of tourism improves the terms of trade, it increases the consumer demand for nontradable goods, raises the relative price of nontradable goods, and transfers resource factors from the tradable sector to the nontradable sector, leading to an appreciation of the real exchange rate and domestic commodity prices, which, in turn, weakens the competitiveness of the tradable sector and reduces the overall welfare of residents [9,10].

Additionally, some studies have shown that tourism development does not have a simple positive or negative linear impact on economic growth, but presents nonlinear characteristics with changes in the level of tourism specialisation. For example, Brau et al. defined 143 countries with an average population of less than 1 million and an average tourism specialisation level higher than 10% from 1980 to 2003 as small countries and used dummy variables for group regression. They found that small countries can achieve rapid economic growth only when the tourism industry is highly specialised [23]. Po and Huang further applied the more advanced panel threshold regression method to study the impact of tourism on economic growth under different tourism specialisation conditions and indicated that inbound tourism specialisation significantly boosted economic growth only when it was below 4.05% or above 4.73% [12]. Based on a cross-sectional threshold regression model, Chiu and Yeh employed three different tourism specialisation indices as threshold variables to examine the impact of tourism on economic growth in 84 countries, and the results confirmed the non-linear relationship between tourism and economic growth, indicating that there are different effects on economic growth in countries with different tourism development conditions [14].

#### 2.1.3. The Environmental Impact of Tourism

The research on the impact of tourism development on the environment includes three viewpoints, namely the “environmental deterioration theory”, “environmental improvement theory”, and “non-linear relationship theory”. Most studies support the first view that the rapid development of tourism is at the cost of environmental pollution and ecological degradation; that is, the development of tourism resources, the construction of tourism projects, and the development of tourism activities will cause problems such as water pollution [44], air pollution [19,45], and soil degradation [46,47], which will adversely affect the environment in tourist destinations. In particular, the increase in carbon dioxide emissions in the environment caused by the energy consumption involved in tourism activities, such as transportation, accommodation, and catering, has become an inevitable problem in the negative impact of tourism-driven environments [16,48,49,50].

However, there are also a few studies that support the second view, claiming that the development of tourism can alleviate greenhouse gas emissions. According to the United Nations Environment Programme, carbon emissions can be greatly reduced by implementing sustainable tourism development plans through the use of cleaner energy and low-emission technologies [51]. Tian et al. investigated the impact of tourism development on carbon emissions in G20 economies from 1995 to 2015 and found that, for every 1% increase in tourism development, pollutant emissions would decrease by 0.05%, indicating that tourism development can be the driving force for reducing carbon emissions [52]. Ahmad and Ma took Asian Tigers as case studies for exploring the role of tourism development in pollutant emissions and its impact mechanism, arguing that tourism development can curb carbon emissions by replacing high-emitting industries and promoting the use of renewable energy [53].

In addition to the above viewpoints, some scholars have concluded that tourism has a non-linear impact on environmental quality. This view is mainly inspired by the environmental Kuznets curve, which argues that, with the improvement of the level of tourism development, pollutant emissions will exhibit an inverted U-shaped curve. For instance, Katircioğlu confirmed the existence of the Singapore tourism-induced EKC hypothesis [54]. The study by Ozturk et al. showed that the EKC assumption between tourism income and the ecological footprint is more prevalent in upper-middle-income countries than in lower-middle-income and lower-income countries [24]. Paramati et al. provided evidence for the EKC hypothesis between tourism and carbon emissions, arguing that the EKC hypothesis for tourism applies in both developed and developing economies [20]. Lv and Xu examined the non-linear impact of tourism on the environment, arguing that, in the early stage of tourism development, tourism’s legal and regulatory systems are not perfect and the relevant supporting policies and regulations are not standardised, leading to a sharp increase in pollution; however, when tourism develops to a certain stage, the laws, regulations, standards, and policies related to tourism are also becoming increasingly improved and well-managed, thereby reducing pollutant emissions [22].

#### 2.1.4. The Impact of Tourism on Green Development

With the deepening of the study on the economic and environmental impacts of tourism and the improvement of the connotation of green development, some studies have begun to bring the economy and environment into a unified framework to explore the impact of tourism on the green economy and green growth, similar to green development. However, the research conclusion has not reached a consensus. 

Some studies claim that the development of tourism is conducive to promoting green growth and achieving the goal of a green economy. For example, Marsiglio deduced that tourism can stimulate emission reduction activities and economic growth incentives by developing a stylised dynamic economic model, positing that a well-planned tourism sector can be an important tool for promoting green growth in multiple developing countries [55]. Pan et al. proposed an interactive framework between tourism and economic, social, cultural, and environmental sustainability, arguing that tourism can contribute to changes in the green economic system [56]. However, other studies hold the opposite view. Holden believes that, while tourism is recognised as a key economic sector in achieving the global transition from a brown to a green economy, he also stresses that the challenge of tourism in achieving the goal of the green economy is the interaction between tourism and the natural environment, which is related to the destruction of the environment by tourists [57]. Law et al. believe that the rapid development of tourism has brought about serious challenges to the green economy, including water shortage and inequity, waste pollution, and loss of biodiversity and habitats [58].

In summary, the academic circle has carried out a relatively systematic study on green development and the external impact of tourism, especially on the aspect of the impact of tourism on the economy and environment, which has accumulated fruitful research results. Nevertheless, most of the existing research focuses on the unilateral impact of tourism on economic growth or environmental pollution. Although a few scholars have incorporated the two directions into a unified research framework, the literature on how tourism affects green development as a research topic is still scarce at this stage, especially research that examines the non-linear impact of tourism on green development. Simultaneously, in terms of research methods, most studies measure the non-linear impact of tourism on the economy or the environment by grouping regression, introducing square terms, or static threshold regression methods. The first two methods often have the problem of subjectivity and high collinearity, while the latter method has difficulty overcoming the endogeneity problem due to the bidirectional causal relationship between the dependent and independent variables. This study aims to close these research gaps in the literature.

### 2.2. Mechanism Analysis and Research Hypothesis

This paper interprets the concept and connotation of green development from a narrow sense; that is, green development aims to promote economic growth while simultaneously responding to resource conservation and environmental pollution reduction so as to achieve the coordination and unity of economic growth and environmental protection [4]. Therefore, mechanism analysis of the impact of tourism on green development should be carried out from two directions: economic growth and environmental protection.

**Figure 1:**
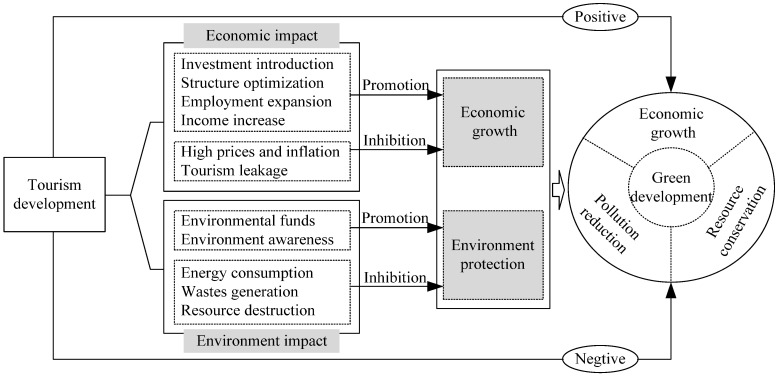
Mechanism of tourism affecting green development.

Theoretically, tourism has both positive and negative impacts on green development, as shown in Figure 1. In terms of economic growth, as an important industry of the national economy, the tourism industry has a high degree of marketisation and strong industry-related driving functions. Therefore, it can promote regional economic growth by attracting investment, adjusting the industrial structure, expanding employment, and increasing national income and local fiscal revenue [5,6,7,37,38]. In terms of environmental protection, the economic benefits brought by the development of tourism can provide financial support for tourist destinations to take necessary environmental protection measures; for example, ticket income and taxes paid by tourism enterprises can be used to pay for the protection and management of natural resources and the ecological environment [59]. Moreover, tourism can also make people more deeply connect with nature and the environment, improve their understanding of the value of the ecological environment, and thus lead to the adoption of environmentally conscious behaviours and activities to protect the environment [59]. Therefore, tourism has a positive impact on regional green development.

Tourism development also has “non-green” problems that are not conducive to economic growth and environmental protection. On the one hand, with tourism development, negative externalities of tourism to the economy have also arisen. The influx of tourists not only increases the consumption of related products in the region but also causes sudden changes in supply and demand, leading to an increase in local prices and inflation. If the economic foundation is weak, resources are limited, or people are insufficient for a tourist destination to meet the development of the sector, then the tourism industry will rely on external investment, personnel, and management [60]. In this case, the diversity of local tourism participation is more likely to be disrupted, and tourism revenue leakage will occur [60]. On the other hand, tourism development is also unfavourable to the destination’s environment. For example, the flow and temporary stays of tourists increase the consumption of resources and energy while leaving behind domestic sewage, rubbish, and waste gas from the use of vehicles, causing water, soil, and air pollution [16,18,19]. In addition, the development of tourist attractions and the large-scale construction of tourism infrastructure, if not properly planned, will cause constructive damage to the surface environment and natural resources.

Based on the above analysis, this paper proposes the following research assumptions:

**Hypothesis** **1.** *The impact of tourism on green development is uncertain, and may be positive or negative*.

From the perspective of the tourism area life cycle and the evolution of the tourism industry, the influence of tourism on green development is not constantly positive or negative, but non-linear. According to the theory of the Tourism Area Life Cycle (TALC), the development of a tourism area system is not static, but undergoes a dynamic evolution process from generation to decline [61]. This process is not only directly related to the healthy development of the local tourism economy, but also the economic and environmental conditions of the tourist areas, which will inevitably change with the tourism development [62].

Specifically, in the initial stage of tourism development, a small number of visits will cause the local tourism industry to show the characteristics of low demand and low supply, and the local economic and environmental conditions will not change significantly [63]. As a result, tourism will have no substantial impact on local green development at this stage. When the tourism industry develops to the growth and mature stages, the demand and supply of tourism will show an increasing trend. The tourism industry has a large scale and can promote economic growth through income effects, employment effects, and industry linkage driving effects, accompanied by a certain threat to the local environment [61,62]. However, the scale expansion of the tourism industry and the improvement of economic benefits in this stage will often bridge the negative impact of the environment. Therefore, compared with the initial stage, the impact of tourism on green development will improve in this stage. With the continuous development of tourism, the large number of tourist visits and the operation of tourism activities will further aggravate the environmental damage, which, in turn, will significantly reduce the attraction of tourist destinations and the profitability of the tourism industry [63]. Once the number of tourists reaches the maximum or exceeds the maximum environmental carrying capacity, the destination’s economic development and ecological environment will become even less sustainable. However, if the private local stakeholders and public administration departments reposition the tourism area, not only emphasising the economic function of the industry, but also taking resource conservation and ecological environmental protection as the important goals of tourism development, the tourism industry will be revived [63]. Therefore, the situation of its negative impact on green development can be improved or even reversed.

Underpinned by an extensive review of the empirical literature and the above statement, this paper proposes the following research assumptions:

**Hypothesis** **2.** *The impact of tourism on green development varies with different levels of tourism development, showing non-linear features*.

## 3. Methodology and Data

### 3.1. The Measurement Method of Green Development

Green development measurement is mainly based on two methods: the green development index [31,32,33] obtained by the comprehensive index system method and the green development efficiency [2,4,30] obtained by the efficiency analysis method. The former is easily interfered with by human factors in the construction of the indicator system, the determination of indicator weights, and the synthesis of indicators, while the green development efficiency, measured based on the efficiency analysis method, is carried out from the perspective of input and output, and the measurement process is more objective. Moreover, the key to green development is improving the green development efficiency, which means driving the transformation of a region using a green development mode with low input, low emissions, and high efficiency [2,4]. Therefore, this study selects the green development efficiency as an indicator to measure green development.

Consistent with a general efficiency evaluation, the green development efficiency is a measure of a region’s ability to achieve the expected output per unit of input cost and is often measured by the data envelopment analysis (DEA) method, which uses the mathematical programming method to measure the relative production efficiency of the decision-making unit to judge whether it is located on the frontier of the production set. Classic DEA models, such as the CCR or BCC models, require the input and output to change in the same proportion when evaluating efficiency and fail to fully consider the slack variable problem of insufficient input and output, which may overestimate the efficiency value of the decision-making units. Furthermore, this model cannot further compare the efficiency of decision-making units on the frontier. To overcome the above problems of the classic DEA model, Tone proposed the SBM model and the superefficient SBM model successively [64,65]. However, different from general efficiency evaluation, the green development efficiency also considers the resource consumption input and bad output in the form of environmental pollution discharge. Consequently, a super-efficient SBM model with undesired outputs was adopted to measure the green development efficiency in this study. The specific form of the model is as follows:

(1) $$\begin{array}{l} \rho =\min\frac{1+\frac{1}{m}\sum_{i=1}^{m}\frac{s_{i}^{x}}{x_{it}}}{1-\frac{1}{s_{1}+s_{2}}\left(\sum_{k=1}^{s_{1}}\frac{s_{k}^{y}}{y_{kt}}+\sum_{l=1}^{s_{2}}\frac{s_{l}^{z}}{y_{lt}}\right)}\\ s.t.\{\begin{array}{c} x_{it}\geq \sum_{j=1,\neq t}^{n}\lambda_{j}x_{j}-s_{i}^{x}\\ y_{kt}\geq \sum_{j=1,\neq t}^{n}\lambda_{j}y_{j}-s_{k}^{y}\\ z_{lt}\geq \sum_{j=1,\neq t}^{n}\lambda_{j}z_{j}-s_{l}^{z}\\ 1-\frac{1}{s_{1}+s_{2}}\left(\sum_{k=1}^{s_{1}}\frac{s_{k}^{y}}{y_{kt}}+\sum_{l=1}^{s_{2}}\frac{s_{l}^{z}}{y_{lt}}\right)\\ s_{i}^{x}\geq 0,s_{k}^{y}\geq 0,s_{l}^{z}\geq 0,\lambda_{j}\geq 0 \end{array} \end{array}$$

where *ρ* represents the green development efficiency; x, y, and z are the input elements, expected output, and undesired output elements, respectively; *m*, *s*_1_, and *s*_2_ represent the number of input, expected output, and undesired output variables, respectively; *t* is the production period; *i*, *k*, and *l* are the decision-making units of the input, desired output, and undesired output, respectively; $s_{i}^{x}$, $s_{k}^{y}$, and $s_{l}^{z}$ are the slack of the input, desired output, and undesired output, respectively; and *λ_j_* is the weight vector. *ρ ≥* 1 indicates that the production decision-making unit is relatively effective, while *ρ* < 1 indicates that the evaluated production decision-making unit is relatively ineffective and has efficiency loss, but the efficiency can be improved by optimising the input, the expected output, and the undesired output.

The calculation process of the green development efficiency involves input indicators and output indicators. Based on relevant studies [2,4,30,66], the input indicators mainly include labour, capital, and energy consumption, and are measured by the number of employees at the end of the year, the capital stock estimated based on the fixed asset investment data of the whole society and the perpetual inventory method, and the electricity consumption of the whole society, respectively. The output indicators include the expected economic benefit output and the undesired environmental pollution output, which are represented by GDP and industrial pollutant (wastewater, sulphur dioxide, and soot) emissions, respectively.

### 3.2. The Models for Tourism’s Impact on Green Development Efficiency

#### 3.2.1. Model Specification

Based on the previous literature review and the mechanism analysis, it can be found that tourism may have both positive and negative effects on green development. Under the reality of China, whether tourism can promote or inhibit green development needs to be tested through empirical models. Therefore, this paper first establishes the following basic model:

(2) $$\ln{GDE}_{it}=\alpha \ln{TDL}_{it}+\lambda_{j}\ln X_{it}+\mu_{i}+\varepsilon_{it}$$

where *GDE* represents the green development efficiency and is the dependent variable in the model; *i* and *t* are the cross-sectional unit and time series, respectively; *TDL* represents the level of tourism development, which is the core independent variable; *λ_j_* represents the parameters to be estimated for the control variable; *X* represents a set of control variables; *μ_i_* is the time-invariant individual effect; and *ε_it_* is the random error term, which is assumed to be independent and identically distributed.

In addition, to investigate whether tourism has a non-linear impact on green development, this study adopts a dynamic panel threshold regression model, which not only determines the threshold value endogenously according to the characteristics of the constraint variables, but also more effectively addresses the potential endogeneity problem [67]. Based on the static threshold panel model proposed by Hansen [68], some scholars have proposed a more advanced dynamic panel threshold model that allows the threshold variables or regressors to be endogenous and uses the GMM estimation method to overcome the endogeneity problem of the model [25,69,70]. We define the dynamic panel threshold model as follows:

(3) $$\begin{array}{ll} \ln{GDE}_{it} & =\alpha_{1}\ln{TDL}_{it}I(\ln{TDL}_{it}\leq \gamma )+\alpha_{2}\ln{TDL}_{it}I(\ln{TDL}_{it}>\gamma )\\ & \begin{array}{c} +\beta \ln \end{array}{GDE}_{i,t-1}+\lambda_{j}\ln X_{it}+\mu_{i}+\varepsilon_{it} \end{array}$$

where *I*(•) is an indicative function. If the expression in parentheses is true, then *I(*•) = 1; otherwise, *I*(•) = 0. *γ* is the threshold value to be estimated, *α*_1_ and *α*_2_ are the parameters to be estimated for the core independent variables under different threshold intervals, and *β* represents the parameters to be estimated for the lag term of the dependent variable. The remaining symbols and variables are mentioned above and will not be repeated here.

Formula (3) is the model form of a single-threshold value, and multiple threshold values may appear in the actual analysis. For this reason, this paper establishes a dynamic multi-threshold regression model of the following form:

(4) $$\begin{array}{ll} \ln{GDE}_{it} & =\alpha_{1}\ln{TDL}_{it}I(\ln{TDL}_{it}\leq \gamma_{1})+\cdots +\alpha_{n}\ln{TDL}_{it}{I(\gamma }_{n-1}<\ln{TDL}_{it}\leq \gamma_{n})\\ & \begin{array}{c} +\alpha_{n+1}\ln{TDL}_{it}I(\ln{TDL}_{it}>\gamma_{n})+\beta \ln \end{array}{GDE}_{i,t-1}+\lambda_{j}\ln X_{it}+\mu_{i}+\varepsilon_{it} \end{array}$$

where the threshold value $\gamma_{1}<\cdots \gamma_{n-1}<\gamma_{n}$ and *α*_1_⋯*α_n_*_+1_ are the parameters to be estimated for the core independent variable in different threshold intervals.

#### 3.2.2. Variable Selection

(1) Dependent variable

This paper selects the green development efficiency (*GDE*) as the dependent variable, which was measured by the super-efficient SBM model with undesired outputs described above.

(2) Core independent variable

The tourism development level (*TDL*), the core independent variable, has been quantified through various economic indicators. The most commonly used indicators include tourism revenues and the number of tourist arrivals [6,13,34]. This paper selects the total tourism revenue index, represented by the sum of the domestic tourism revenue and inbound tourism revenue converted into RMB according to the average exchange rate of the year, to measure the variable [71]. In addition, this paper also uses another important indicator to measure the *TDL*, namely the number of total tourist arrivals, to test the robustness.

(3) Threshold variable

As mentioned in the previous theoretical analysis, the impact of tourism on green development may be non-linear. To examine the non-linear impact of tourism on green development, the *TDL* is also incorporated into the model as a threshold variable.

(4) Control variables

To accurately analyse the impact of tourism on green development and avoid model estimation errors caused by missing variables, based on previous research results, this paper selects the following key variables that affect green development to control: ① Economic development level (*EDL*), which is the economic foundation of regional green development, expressed by GDP per capita [2]. ② Technological innovation (*TI*), which is an effective way to improve green development, characterised by the proportion of science and technology expenditure in GDP [4]. ③ Human capital (*HC*), which often affects economic growth and environmental quality by promoting technological progress and improving people’s environmental awareness, so it can provide intellectual support for regional green development [66]. This paper uses the number of college students in every ten thousand people to indicate *HC*. ④ Industrial structure (*IS*), which has a great impact on green development. Different types of industries have different impacts on regional energy consumption and environmental pollution, while the development of secondary industry often depends on resource and energy consumption [1]. Referring to Dong et al., this study uses the proportion of secondary industry output in GDP to measure the *IS* [58]. ⑤ Foreign direct investment (*FDI*), which can promote regional green development through technology spillovers and demonstration effects and is characterised by the proportion of foreign direct investment in GDP [2]. ⑥ Environmental regulation (*ER*), which is an effective means for the government to address environmental pollution and plays an indispensable role in improving green development [72]. This paper reflects the *ER* in terms of the removal rate of industrial sulphur dioxide. 

In this paper, all of the above variables are logarithmically treated in model setting to eliminate the influence of heteroscedasticity. The descriptive statistical results in the logarithmic form of the relevant variables are shown in Table 1.

### 3.3. Data

In view of the continuity of space and the availability of data, this study excluded cities with significant missing data and adjusted the administrative divisions during the study period; it finally selected 284 cities at the prefecture level and above in mainland China as research samples. Among them, tourism-related data were obtained from the CEIC China Economic Database, https://insights.ceicdata.com/ (accessed on 2 May 2021), while data related to the input and output indicators to measure the green development efficiency and data related to the control variables that affect the green development efficiency were all from the *China Urban Statistical Yearbooks* from 2003 to 2019 [73]. For individual missing data, interpolation was used as a supplement.

## 4. Empirical Results

### 4.1. Analysis of Measurement Results of Green Development Efficiency

**Figure 2:**
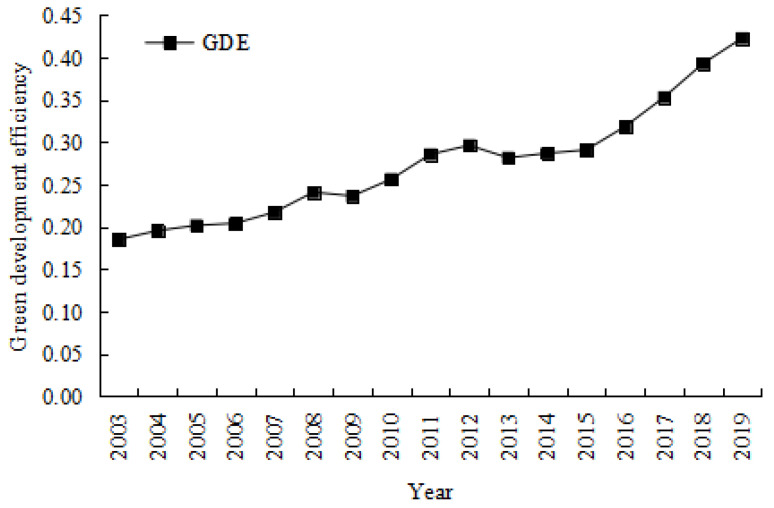
Evolution trend of the green development efficiency in China (2003–2019).

Based on the above super-efficiency SBM with undesirable outputs, this study used MaxDea to measure the green development efficiency. Figure 2 shows the evolution trend of the average green development efficiency of 284 cities at the prefecture level and above in China from 2003 to 2019.

According to Figure 2, China’s green development efficiency showed a fluctuating and rising trend, with obvious stage characteristics. From 2003 to 2008, China’s green development efficiency showed a steady upward trend. At this stage, driven by the demographic dividend and large-scale investment and construction, China’s economy showed a trend of rapid growth. However, the resulting environmental pollution also become a key factor that curbed the rapid improvement of the green development efficiency. From 2009 to 2012, China’s green development efficiency improved rapidly. Under the influence of the global financial crisis and the near-full capacity of heavy industries supporting China’s economic growth, the country has rapidly improved its green development efficiency by eliminating overcapacity heavy industries and investing in emerging technology industries and services. Since 2013, China has entered a critical period of economic transformation, emphasising economic quality improvement and focusing on green development and ecological civilisation construction. Therefore, compared with the previous two stages, China’s green development efficiency has improved faster. 

In general, during the sample period, China’s green development efficiency showed an upward trend but eventually entered a low level. The reason for this is that China’s economy has accumulated serious ecological and environmental problems in the process of long-term rapid growth. In this regard, the Chinese government has successively put forward the concepts of circular economy, low-carbon economy, ecological civilisation, and green development, promoting the benign interaction between economic development and the ecological environment. However, due to the path dependence of the traditional extensive development mode, green development still faces severe challenges.

### 4.2. Linear Analysis of Tourism’s Impact on Green Development

#### 4.2.1. Benchmark Regression

We first used a panel data model to verify the impact of tourism on the green development efficiency. Whether to use a mixed regression model that requires each individual in the sample to have the exact same regression equation or to choose a fixed-effects model or a random-effects model that considers heterogeneity among individuals is a fundamental issue when dealing with panel data. First, the F test and the LM test provided by Breusch and Pagan were chosen to verify whether there was an individual effect of heterogeneity among individuals. If the test results indicated that a panel data model with individual effects needed to be applied, the Hausman test was further used to determine whether to choose a fixed-effects model or a random-effects model.

Table 2 shows the results of the discriminant test of the panel data model. Both the F test and the B-P LM test passed the significance test at the 1% level, strongly rejecting the null hypothesis that there was no individual effect, and both the fixed-effects model and the random-effects model were better than the mixed regression model. Furthermore, the Hausman test was also statistically significant, indicating that the fixed-effects model was more effective than the random-effects model. Therefore, this paper analyses the impact of tourism on the green development efficiency based on the estimation results of the fixed-effects model in Table 3.

The estimated coefficient of the tourism development level was significantly positive at the 1% level, and for every 1% increase in the tourism development level, the green development efficiency increased by 0.044%, indicating that tourism development could effectively promote the improvement of China’s green development efficiency. From the results of the control variables, all variables passed the significance test at the 1% level. Among them, the regression coefficients of *EDL*, *TI*, *HC*, and *ER* were all significantly positive, indicating that the improvement in the economic development level, the improvement in the technological innovation ability and human capital, and the strengthening of environmental regulation can promote the improvement of the green development efficiency. However, the estimated coefficients of *IS* and *FDI* were negative, which means that the industrial structure and foreign direct investment had an adverse impact on the green development efficiency. The reason is that, from the perspective of industrial structure, as China’s economic growth is excessively dependent on secondary industry, especially the heavy chemical industry with high energy consumption and pollution emissions, the industrial structure of heavy industry is not conducive to the improvement of the green development efficiency [66,74]. As far as foreign direct investment is concerned, China’s long-term basic line of economic construction has enabled local governments to promote economic development by introducing foreign investment. Various regions tended to relax their environmental control standards to attract more foreign capital inflows, speeding up the use of natural resources and the production of pollution-intensive products, thus aggravating environmental pollution and making China a “pollution paradise” for developed countries [75].

#### 4.2.2. Robustness Testing

To test the robustness of the model estimation results, this paper re-estimates the model by replacing the core explanatory variables, that is, selecting the total number of tourist receptions as a proxy variable for the level of tourism development. The robustness of the model’s estimation results was also tested by changing the sample size. The sample data in this paper contained 284 cities at the prefecture level and above, including municipalities directly under the Central Government, provincial capital cities, and ordinary prefecture-level cities. The special administrative status may render the municipalities directly under the Central Government and provincial capital cities incomparable with ordinary prefecture-level cities. In view of this, this study deleted the data of municipalities directly under the Central Government and provincial capital cities, and used the remaining 254 samples for regression.

According to the results of the F test, B-P LM test, and Hausman test in Table 4, the fixed-effects model was employed as the optimal model for the robustness test, and we found that, from the perspective of the estimated coefficient size, sign, and significance of the variables, the sign of the impact of tourism on China’s green development efficiency was still significantly positive, and the parameter values did not change much. The sign and significance of the influence of the remaining control variables were consistent with the results of the benchmark regression model, indicating that the estimated results of the model were robust.

### 4.3. Non-Linear Analysis of Tourism’s Impact on Green Development

#### 4.3.1. Threshold Estimation and Threshold Effect Test

Although the above analysis verifies the positive impact of tourism on the green development efficiency, considering the life cycle of tourism destinations and the nonequilibrium characteristics of tourism development levels, tourism may have a differentiated impact on the green development efficiency, and the estimation results based on linear panel data models may mask such non-linear characteristics. Therefore, based on the dynamic panel threshold regression model constructed in the methods section, this paper empirically explores the differential and complex mechanism of tourism affecting green development under the threshold of the tourism development level.

Before estimating the dynamic panel threshold regression model, it is necessary to clarify the estimated value of the threshold parameters and conduct a self-sampling test on the threshold effect. The results are shown in Table 5. In the single-threshold model and the double-threshold model, the threshold values in the logarithmic form of the tourism development level were 7.157 and 11.109, respectively, and the threshold effects were all significant at the 1% level, which meant that, in the three different intervals divided by the two threshold values, there were significant differences in the impact of tourism on the green development efficiency. In the triple threshold model, however, the threshold effect was not significant, and the estimation results of the double-threshold model should, therefore, be used as the analysis basis in future research.

#### 4.3.2. Estimation Results of the Threshold Effect Model

Combined with the above threshold estimation and threshold effect test results and based on the dynamic panel threshold regression model method, this study further uses the systematic GMM estimation method to estimate the dynamic panel threshold effect model. Table 6 reports a series of tests to verify the effectiveness of the systematic GMM estimation method and the regression results of the dynamic double-threshold model.

The *p* values of AR(1) and AR(2) were 0.014 and 0.360, respectively, indicating that there was a first-order autocorrelation in the difference in the disturbance term, but there was no second-order autocorrelation, which supports the null hypothesis that the disturbance term of the regression model had no autocorrelation. Furthermore, through the Sargan test, it was determined that the test result was not significant, and there was no overidentification problem, verifying the validity of the system GMM estimation. The estimation results of the dynamic panel threshold model show that, in the three different intervals in which the logarithm of the total tourism income was lower than 7.157, between 7.157 and 11.109, and higher than 11.109, the influence coefficients of tourism on the green economic efficiency were 0.033, 0.046, and 0.071, respectively, and they all passed the significance test at the 1% level. This result suggests that the contribution of tourism to the green development efficiency in China was non-linear, meaning that, as the level of tourism development increased beyond a certain threshold, the contribution to the green development efficiency would increase.

#### 4.3.3. Robustness Testing

To ensure the robustness of the above model estimation results, we still followed the practice of linear analysis in the previous section by replacing the core explanatory variables and changing the research samples to test the robustness of the dynamic panel threshold model. Table 7 shows the threshold estimation and threshold effect test results of the dynamic panel threshold model under these two robustness testing methods. The threshold estimates of the tourism development level under the two robustness tests were very close to the threshold estimates in Table 5, and the threshold effect test results showed that the single-threshold and double-threshold effects were statistically significant, while the triple-threshold effect was not significant, indicating that the double-threshold effect model should be used for analysis, which is also consistent with the results of the benchmark model. This also verifies the robustness of our estimation results to a certain extent.

Table 8 further shows the estimation results of the dynamic panel double-threshold model based on the two robustness tests. From the estimation method of the model, according to the results of AR(1), AR(2), and the *p* value of the Sargan test, the model estimation results obtained by the systematic GMM estimation method were shown to be valid in both robustness testing methods. In terms of the estimated coefficients of the variables, the results of the estimated coefficient of the tourism development level showed that, with the improvement of the tourism development level, after exceeding a certain threshold value, the influence coefficient of tourism on the green development efficiency was significantly positive and improved, which is consistent with the estimation results of the benchmark model in Table 6. Moreover, the sign of the influence of the green development efficiency lagging by one period and other control variables remained unchanged, and the estimated coefficient and significance did not change significantly. In conclusion, whether it was replacing explanatory variables or deleting some incomparable research samples, the estimation results of the dynamic panel threshold regression model were robust, and more reliable conclusions could be drawn based on the results.

## 5. Discussion

As a derivative concept of sustainable development that gives consideration to both economic growth and environmental protection, green development is the foundation of economic transformation in China and the world. The realisation of the concept of green development requires the support of the corresponding green industry system. Tourism has the attributes of a green industry because of its strong correlation and environmental friendliness. There has been widespread concern about the externalities of tourism on the destination’s economy and environment [5,18], as well as tourism development under the framework of sustainable development goals [76]. Nevertheless, few studies have focused on the questions of “Does tourism affect green development? If so, does this impact have non-linear threshold characteristics?” This paper aims to answer these two questions and fill in the gaps in existing research.

The analysis results of the green development efficiency show that China’s green development efficiency improved during the study period. However, restricted by the world’s industrial division pattern and productivity technology level, China’s extensive development mode has not been effectively transformed, and the ecological environment is still grim. As a result, the green development efficiency has never reached a high level. Moreover, the results of the linear impact analysis model also found that the industrial structure with high energy consumption and high pollution was also an important factor restraining the improvement of China’s green development efficiency. Therefore, to promote the positive interaction between China’s economic development and the ecological environment, and promote green development, it is necessary to change the production mode, develop green industries, and form an industrial structure that saves energy and resources, and protects the ecological environment.

Based on the empirical findings in the previous section, this paper demonstrates that tourism can contribute to the regional green development in China, in line with the views of scholars, such as Marsiglio [55] and Pan et al. [56]. Over 40 years since the reform and opening up, China’s tourism industry has flourished in the wave of marketisation. It is a strategic pillar industry of China’s national economy and a strong engine for economic transformation and upgrading in various regions, as it has made important contributions to generating foreign exchange earnings, increasing tax revenue and jobs, improving infrastructure, and driving poverty-stricken areas out of poverty. Moreover, as tourism mainly satisfies the consumption needs of tourists for excursions, it is characterised by low resource consumption and low environmental pollution. In addition, in recent years, under the premise of advocating for and practising the concept of green tourism consumption, specific countermeasures have been taken across China in the tourism sector, such as the expansion of bioenergy, energy-efficient vehicles for transportation, pollution taxes, green innovative technologies, etc. [77]. Tourism has thus become a vehicle for the construction of ecological civilisation and green development in China.

Further, this study also found that the contribution of tourism to China’s green development efficiency will intensify as the level of tourism development rises to a certain level. Combining the theoretical basis of the Tourism Area Life Cycle with the realities of China’s situation, the development objectives of the tourism industry are often focused on the pursuit of industry scale and economic benefits at a low level of development. Although the increase in tourism revenue has brought economic gains to destination development, tourism development is resource-driven and capital-driven, and this crude development model is unsustainable. The blind and rapid expansion of tourism has resulted in low efficiency of resource allocation and excess production capacity of primary tourism products, resulting in waste of resources and environmental pollution, to a certain extent. Therefore, the contribution of tourism to the green development efficiency within this phase is not outstanding. As tourism continues to grow and enter a higher level of development, the tourism industry focuses on both economic attributes and the enhancement of overall benefits, such as social and environmental benefits, thus strengthening the contribution of tourism to green development. On the one hand, through the transformation of the traditional tourism industry and the integration of tourism with other industries, the tourism industry and products are constantly being innovated and enriched, and the tourism industry structure is being optimised and upgraded. As a result, tourism has become an important driving force in boosting consumption growth and contributing to high-quality economic development. On the other hand, to achieve healthy and sustainable tourism development, local governments across China have adopted stricter environmental policies, as well as scientific planning schemes and management methods. Tourism-related sectors are also promoting the improvement of environmental quality by adopting clean energy and technologies, such as green tourist hotel programs, new energy transportation equipment, etc.

This study explores the impact of tourism on green development from both theoretical and empirical perspectives, enriching the research on tourism externalities and the research framework of its effects on green development. In addition, the empirical analysis of a series of econometric models can effectively identify the role of tourism in China’s green development in order to provide a reference for the implementation of China’s green development strategy and the rational development of the tourism industry. However, there are still some limitations. First, due to data limitations, the research time scale did not include the time period since the COVID-19 epidemic. Future research will explore the impact of tourism on green development during the epidemic. Second, this paper explores the impact of tourism on green development at an urban scale, but the impact of tourism on green development may be different at different spatial scales. Future research will be extended to exploring this issue at the provincial scale in China and even at the global level of individual countries. Third, this paper only explores the non-linear relationship between tourism and the green development efficiency through the lens of heterogeneity in the stage of tourism development in a destination, ignoring the heterogeneity in resource endowment, economic status, and even city size between destinations. Future research will comprehensively examine the non-linear impact of tourism on green development under multiple heterogeneities.

## 6. Conclusions

Based on a panel dataset of 284 Chinese cities at the prefecture level and above from 2003 to 2019, we took the green development efficiency measured by the super-efficient SBM model with undesired output as a proxy indicator of green development and applied the panel data regression model and dynamic panel threshold regression model to comprehensively investigate the impact and non-linear characteristics of tourism on green development. The empirical analysis results confirmed that the development of tourism has become a driver of the green transformation and development of China’s economy and provided empirical evidence for the non-linear impact of tourism on green development. Specifically, tourism development can promote the improvement of the regional green development efficiency in China, and, compared with cases where the level of tourism development is still low, the positive effect of tourism on the efficiency of green development is stronger at higher levels of tourism development.

## Figures and Tables

**Table 1:** Descriptive statistics of main variables.

Variables	Observation	Mean	Std. Dev	Min	Max
ln*GDE*	4828	−1.431	0.548	−6.112	0.154
ln*TDL*	4828	9.083	1.610	2.197	13.341
ln*EDL*	4828	10.204	0.824	7.545	12.281
ln*TI*	4828	−2.180	1.102	−6.479	4.309
ln*HC*	4828	4.373	1.240	−1.849	7.288
ln*IS*	4828	3.829	0.260	2.197	4.511
ln*FDI*	4828	0.071	1.474	−5.107	6.203
ln*ER*	4828	0.465	0.546	−2.764	2.168

**Table 2:** Discriminant tests for panel data models.

	F Test	B-P LM Test	Hausman Test
Statistical value	47.084 ***	5141.726 ***	145.209 ***
*p* value	0.000	0.000	0.000

Note: *** represents significance at the 1% level.

**Table 3:** Regression results of linear panel data fixed-effects model.

Variables	Coefficient	T-Statistic	*p* Value
ln*TDL*	0.044 ***	4.499	0.000
ln*EDL*	0.457 ***	24.815	0.000
ln*TI*	0.078 ***	8.970	0.000
ln*HC*	0.086 ***	7.885	0.000
ln*IS*	−0.461 ***	−17.002	0.000
ln*FDI*	−0.054 ***	−11.269	0.000
ln*ER*	0.065 ***	5.797	0.000
*Cons*	−7.506 ***	−20.068	0.000

Note: *** represents significance at the 1% level.

**Table 4:** Robustness test of the model estimation results.

Variables	Variable Replacement			Sample Replacement		
	Coefficient	T-Statistic	*p* Value	Coefficient	T-Statistic	*p* Value
ln*TDL*	0.030 ***	6.152	0.000	0.056 ***	5.712	0.000
ln*EDL*	0.490 ***	27.784	0.000	0.411 ***	21.862	0.000
ln*TI*	0.078 ***	8.583	0.000	0.069 ***	8.386	0.000
ln*HC*	0.085 ***	7.736	0.000	0.068 ***	6.256	0.000
ln*IS*	−0483 ***	−17.680	0.000	−0.351 ***	−12.147	0.000
ln*FDI*	−0.052 ***	−11.329	0.000	−0.053 ***	−9.910	0.000
ln*ER*	0.067 ***	6.001	0.000	0.064 ***	5.583	0.000
*Cons*	−7.850 ***	−21.385	0.000	−6.361 ***	−16.523	0.000
F test	46.961 ***			46.034 ***		
B-P LM test	5128.153 ***			4504.428 ***		
Hausman test	151.036 ***			124.670 ***		

Note: *** represents significance at the 1% level.

**Table 5:** Results of threshold value estimation and significance test of the threshold effect.

Threshold Variable	Threshold Type	Threshold Value	95% Confidence Interval	F-Statistic	*p* Value
*TDL*	Single	7.157	[7.093, 7.172]	174.694 ***	0.000
	Double	11.109	[11.080, 11.132]	95.933 ***	0.000
	Triple	12.103	[12.052, 12.150]	32.364	0.567

Notes: The F-statistic, *p* value, and 95% confidence interval in the table are based on the results of repeated sampling 400 times by the bootstrap method. *** represents significance at the 1% levels.

**Table 6:** Estimation results of dynamic panel threshold regression models.

Variables	Coefficient	Standard Error	*p* Value
ln*TDL* (ln*TDL* ≤ 7.157)	0.033 ***	0.013	0.001
ln*TDL* (7.157 < ln*TDL* ≤ 11.109)	0.044 ***	0.010	0.000
ln*TDL* (ln*TDL* > 11.109)	0.071 ***	0.011	0.000
ln*GDE_i,t−1_*	0.528 ***	0.150	0.000
ln*EDL*	0.220 ***	0.054	0.001
ln*TI*	0.058 ***	0.013	0.000
ln*HC*	0.092 ***	0.024	0.000
ln*IS*	−0.198 ***	0.015	0.000
ln*FDI*	−0.055 ***	0.057	0.007
ln*ER*	0.071 ***	0.031	0.023
*Cons*	−1.466 ***	0.552	0.008
AR(1)	-	-	0.013
AR(2)	-	-	0.360
Sargan test	-	-	0.703

Note: *** represents significance at the 1% level. “-” indicates that the item is not involved.

**Table 7:** Robustness test of threshold value estimation and threshold effect test.

Method Selection	Threshold Type	Threshold Value	95% Confidence Interval	F-Statistic	*p* Value
Variable replacement	Single	7.754	[7.664, 7.770]	184.273 ***	0.000
	Double	10.779	[10.768, 10.792]	63.935 ***	0.010
	Triple	11.717	[11.681, 11.771]	25.282	0.573
Sample replacement	Single	7.130	[7.055, 7.152]	146.840 ***	0.000
	Double	10.546	[10.531, 10.563]	35.652 *	0.063
	Triple	11.105	[10.989, 11.131]	20.485	0.613

Note: The F-statistic, *p* value, and 95% confidence interval in the table are based on the results of repeated sampling 400 times by the bootstrap method. *** and * represent significance at the 1% and 10% levels, respectively.

**Table 8:** Robustness test of estimation results of dynamic panel threshold regression model.

Variables	Variable Replacement			Sample Replacement		
	Coefficient	Standard Error	*p* Value	Coefficient	Standard Error	*p* Value
ln*TDL* (ln*TDL* ≤ *γ*_1_)	0.048 ***	0.011	0.002	0.056 ***	0.010	0.000
ln*TDL* (*γ*_1_ < lnTDL ≤ *γ*_2_)	0.060 ***	0.010	0.000	0.069 ***	0.009	0.000
ln*TDL* (ln*TDL* > *γ*_2_)	0.079 ***	0.011	0.000	0.082 ***	0.009	0.000
ln*GDE_i,t−_*_1_	0.516 ***	0.147	0.000	0.562 ***	0.153	0.000
ln*EDL*	0.167 ***	0.062	0.007	0.208 ***	0.047	0.006
ln*TI*	0.055 ***	0.013	0.000	0.062 ***	0.015	0.000
ln*HC*	0.082 ***	0.022	0.000	0.097 **	0.023	0.014
ln*IS*	−0.146 ***	0.055	0.006	−0.179 **	0.074	0.015
ln*FDI*	−0.067 ***	0.053	0.010	−0.059 ***	0.056	0.004
ln*ER*	0.086 ***	0.030	0.004	0.075 **	0.031	0.015
*Cons*	−1.782 ***	0.671	0.009	−1.282 ***	0.495	0.010
AR(1)	-	-	0.015	-	-	0.018
AR(2)	-	-	0.363	-	-	0.365
Sargan test	-	-	0.681	-	-	0.539
*γ*_1_	7.754			7.130		
*γ*_2_	11.717			11.105		

Note: *** and ** represent significance at the 1% and 5% levels, respectively. “-” indicates that the item is not involved.

## Data Availability

Not applicable.
